# A new patient navigation model of care to support older adults in transitions of care: Key considerations for implementation for policy-makers and health leaders

**DOI:** 10.1177/08404704241288458

**Published:** 2024-10-06

**Authors:** Grace Liu, Kristina Kokorelias, Amanda Knoepfli, Tracey DasGupta, Naomi Ziegler, Emma Elliot, Sara J. T. Guilcher, Sander L. Hitzig

**Affiliations:** 1 282299 Sunnybrook Research Institute, Toronto, Ontario, Canada.; 2 518775 Sinai Health and the University Health Network, Toronto, Ontario, Canada.; 3 7938 University of Toronto, Toronto, Ontario, Canada.; 4 SPRINT Senior Care, Toronto, Ontario, Canada.; 5 142153 Sunnybrook Health Sciences Centre, Toronto, Ontario, Canada.

## Abstract

A Patient Navigation (PN) Model of Care was introduced in a large metropolitan hospital in Ontario (Canada) to support transitions in care for older adults in 2019. The patient navigator is a community social worker or “community transitional lead” embedded in the hospital’s in care teams to assist with discharge planning and provide follow-up care to older adults, their families, and/or care partners for up to 90 days. Initially, the PN program supported acute care patients and has since expanded in the Emergency Department and Reactivation Care Centre. In this cohort retrospective observational study, we described the new PN Model of Care by analyzing the clinical notes collected by the patient navigator. This article provides preliminary insights for health leaders who are interested in implementing this novel PN model to improve transitions of care in a hospital setting. Funding was provided by the SLAIGHT Family Foundation.

## Introduction

Patient Navigation (PN) models of care aim to support patients, their families, and/or care partners in navigating the healthcare system, which is often fragmented and complex. First introduced in the early 1990s, PN has expanded and become a recognized healthcare service delivery model^1^ that has been applied across the entire healthcare continuum, which includes prevention, detection, diagnosis, treatment, and end-of-life care.^2^ Specific areas for PN have been developed for conditions (e.g., cancer care), critical care points (e.g., transitional care), and various populations (e.g., mental health and older adults).^3^ Overall, PN is a person-centred healthcare service delivery model to create a seamless flow for patients as they journey through the care continuum.^1^ The definition of “person-centred approach” means focusing on the elements of care, support and treatment that matter most to the patient, their family, and carers.^4^

The role of a patient navigator is typically described as a function that is assigned to a qualified healthcare professional as a component of their role, among multiple existing tasks.^5,6^ This function is most effectively carried out through one-on-one relationships between the patient navigator and the patient,^1^ where the navigator engages with patients, their families, and/or care partners to identify barriers to accessing care, provide referrals to services, facilitate transitions of care, and promote self-management.^3,7^ There is evidence indicating that PN programs can contribute to improved access and continuity of care.^3^ However, the evidence to support the effectiveness of PN programs on patient outcomes is limited.^7-10^

### Transitions of care from hospital to home

One population that may benefit from PN are older adults with complex health and social care needs,^11^ as many older adult patients report sub-optimal outcomes while transitioning from hospital to home.^12^ Many patients often experience system challenges when discharging from hospital to home resulting in repeat Emergency Department (ED) visits^13^ or delayed discharges or Alternative Level of Care (ALC).^14^ In Canada, the term ALC is used to identify hospital beds that are occupied by patients who could be cared for elsewhere due to a mismatch between the patients’ care needs and the services provided in that care setting.^15^ In some cases, patients designated as ALC remain in acute care as they are unable to be discharged home, due to capacity issues in other parts of the healthcare system, such as a lack of home and community care support services available or long-term care beds.^16^ Since over 17% of patients of all acute care bed-days in Canada in 2020-2021 were designated as ALC,^17^ transitional care interventions should be implemented across sectors to link patients to appropriate community-based services^18^ to help patients while they are in the hospital and provide follow-up support.^19^

### Background

Due to challenges faced by older adults, Sunnybrook Hospital, a major metropolitan acute care hospital, partnered with SPRINT Senior Care, which is a community agency to improve the transitions of care to create a PN program. The PN program was introduced in 2019 to improve the continuity of care across care transitions for older adult patients with complex health and social care needs. In this study, the patient navigator is a social worker, called “community transitional lead” who is employed by a community-agency partner embedded in the hospital teams to provide follow-up support to patients, their families, and/or care partners. Upon referral, the patient navigator would assess the patients’ needs and assist patients, their families, and/or care partners as they transition from hospital to community for up to 90 days post-discharge.

Given the novelty of the PN program in a hospital setting, we used an implementation science approach to facilitate the implementation of this new care model.^20^ This included using implementation science to inform a qualitative evaluation (phase 1), which included the design of our interview guides and analysis of data, and helped to establish a quantitative phase (phase 2). The purpose of phase 1 was to explore the experiences of older adults (family caregivers) and healthcare providers (hospital and community) with PN program through semi-structured interviews. Qualitative findings from an earlier iteration of the PN program at our site (phase 1) found that older adults, their families, and/or care partners noted high rates of satisfaction with the PN program,^21^ and identified important considerations for implementing this new model of care.^22^ In phase 2, we summarized the patient navigators’ clinical tracking sheets to better understand the population served and related outcomes. The purpose of phase 2 was to quantitatively evaluate the PN Model of Care by detailing patients’ socio-demographic profile and patient navigators’ scope of practice, which included detailing services provided and patient outcomes. This article provides preliminary insights to inform policy-makers and health leaders who are interested in implementing a PN Model of Care in a hospital setting.

## Methods

The study used a retrospective cohort observational design where data from three cohort groups of patients assigned to the patient navigators’ caseloads were evaluated. We obtained clinical tracking sheets collected by three patient navigators’ who work in the hospital’s Acute Care (AC), Emergency Department, and Reactivation Care Centre (RCC) units.

### Setting and participants

The inclusion criteria were patients referred and assigned to the PN’s caseloads: (1) AC cohort (November 2019 to October 2021), (2) ED cohort (November 2020 to October 2021), and (3) RCC cohort (November 2021 to October 2022). For the data analysis, we included first-time patients who were assigned to the PN’s caseload. Patients who were followed for ≤3 days in the AC and RCC cohorts and for ≤7 days in the ED cohort were categorized as consultations since they were not formally added to the PN’s caseload. Consultations are characterized as the initial PN contact whereby a patient’s suitability for the PN program is assessed or where only a brief interaction was required (e.g., to provide information about available services) with no sustained follow-up. Patients assigned to the PN caseload received follow-up care determined by the PN for up to 90 days or longer (if deemed necessary).

### Data collection and analysis

The data collected included: (1) patient socio-demographics profile (i.e., chronological age and sex); (2) patient navigator scope of practice (i.e., types of interventions); (3) service (i.e., response time and follow-up duration); and (4) outcomes (i.e., post-discharge location). Data were then screened for completeness and reviewed by the first author and senior-responsible author. Open-ended descriptive detailing types of interventions were coded and then frequencies and descriptive statistics were used. The summary results were presented and validated by the PN management team.

## Results

In this study, 90 patients were assigned to the AC patient navigator’s caseload, 46 to the ED patient navigator’s caseload, and 70 to the RCC patient navigator’s caseload. The results include socio-demographic profiles, scope of practice, service, and outcomes of patients who were seen by the patient navigators.

### Socio-demographic profiles

In the three cohorts, the mean age was 79 to 80 and median age was 79 to 81 which ranged from 55 to 98 years of age. The ratio for women ranged from 54% to 70%, compared to men from 28% to 46%.

### Patient navigators’ scope of practice

In terms of the patient navigators scope of practice, the patient navigators may have provided one or more interventions. In the three cohorts, the patient navigators were involved with discharge planning (AC = 61%, ED = 94%, RCC = 44%), service connections (AC = 66%, ED = 80%, RCC = 67%), provider connections (AC = 59%, ED = 37%, RCC = 24%), housing alternatives (AC = 31%, ED = 9%, RCC = 21%), instrumental activities of daily living (AC = 16%, ED = 30%, RCC = 14%), social concerns (AC = 11%, ED = 24%, RCC = 21%), and family/care partner concerns (AC = 31%, ED = 26%, RCC = 16%).

### Patient navigators’ program and outcomes

For the three cohorts, the response time the patient navigators contacted the patients, their families, and/or care partners were as followed: on the same day (AC = 30%, ED = 52%, RCC = 13%), within 7 days (AC = 52%, ED = 39%, RCC = 60%), from 8 to 14 days (AC = 8%, ED = 4%, RC = 6%) or ≥15 days (AC = 6%, ED = 4%, RCC = 9%). The PN program length of service was 49 days (median) and 74 (mean) days in the AC cohort ranging from 5 to 344 days, 67 days (median), and 92 days (mean) in the ED cohort ranging from 10 to 350 days, and 58 days (median) and 66 days (mean) in the RCC cohort ranging from 5 to 241 days.

Upon discharge from PN program, most patients were either discharged home or to a supportive/retirement home setting (AC = 66%, ED = 83%, RCC = 76%). Some of the patients were discharged to an alternative setting (i.e., acute care, rehabilitation, transitional care, long-term care, or palliative care) (AC = 23%, ED = 9%, RCC = 16%). The remaining cases were considered “other,” which were unknown, discharged to a shelter, or deceased (AC = 11%, ED = 9%, RCC = 9%). Cases considered loss to follow-up were “unknown.”

## Discussion

In this retrospective cohort observational design study, we used the patient navigators clinical notes to describe the patients' socio-demographic profiles, patient navigator scope of practice, services, and outcomes. Specifically, we summarized the patient navigators’ caseloads in three cohorts in AC, ED, and RCC to better understand the PN Model of Care in a hospital setting. The findings indicated that the patient navigators played an active role in helping predominantly female older adults in their eighties to support the hospital’s teams in discharge planning and facilitating transitions of care in AC, ED, and RCC.

According to Statistics Canada (2024), older Canadian women face challenges accessing medical specialities and non-emergency tests compared to older men.^22^ In particular, the majority of patients helped by the patient navigators in the ED were women (70%) in this study.^23^ When comparing the emergency visits for older adults (age ≥60 years) from the Canadian Institute for Health Information (fiscal 2020 and 2021), the ratio of women to men was approximately equal (women = 51% and men = 49%). Hence, it is important to better understand why a higher proportion of women were supported by the patient navigator, which could be related to other intersecting socioeconomic factors impacting women compared to men which needs to be explored (i.e., ethnicity/race, immigrant, live alone, and low-income). Since older women (21%) are more likely to be lonely than men (14%)^24^ the PN program may benefit those who live alone.

The main scope of practice of patient navigators in a hospital setting was discharge planning and assisting with service and provider connections. Since the patient navigators are social workers who are employed by a community-agency partner, they were involved with discussing housing alternatives and addressing instrumental activities of daily living needs with the patients, their families, and/or care partners. Given that lower-income seniors experience challenges as they transition from hospital to home^25^ this PN Model of Care with a social worker may be more suitable in addressing housing needs. The patient navigators also provided counselling to support patients, their families and/or care partners cope with stress or burnout.

Since the patient navigators were embedded in hospital, they worked closely with the care teams and were responsive to the needs of the older adult patients, their families, and/or care partners in coordinating discharge plans. The patient navigators were also able to contact the patients within the same day as the referral. For example, the ED patient navigator saw 52% of the patients on the same day. The findings in this research align with the qualitative study in the same hospital during the initial implementation of PN program. In the interview findings, patients and healthcare providers in acute care and community care revealed that the patient navigators facilitated hospital-to-home transitions for older adults with complex needs.^10^ Further, the key informants also reported that having a single designated healthcare provider, such as a patient navigator, can improve patient experience and satisfaction.^20^

In the current literature, there are no standards for PN program duration for this population, as the most effective transitional care strategies for older adults remain unknown.^12^ In this study, the duration for PN program ranged widely from 5 to 350 days, although the median was 49 days in the AC cohort, 58 days in the RCC cohort, and 67 days in the ED cohort. A finding that is relevant to PN program planning is that patients in the ED cohort were actually followed-up for a longer period compared to the AC or RCC cohorts in this study. Transitional care interventions are recommended at pre- and post-discharge to prevent ED visits by older adults; however, there is a need for more evidence-based research regarding transitional care interventions in minimizing ED visits.^12^

As per the clinical notes, in some cases, the patient navigators provided follow-up care until the patient was discharged to another care provider (i.e., case manager or primary care). Since the PN program is novel, patient navigators were providing follow-up care to the patients, their families, and/or care partners as long as needed. This aligns with Kokorelias and colleagues’ (2022) work, who pointed out that PN programs should be considered for older adults with complex care needs as soon as they are assigned to the hospital and for as long as needed.^20^

### Limitations

In the patient profiles, we only collected information on age and sex and did not obtain details about other social determinant factors (i.e., ethnic/race, live alone, low-income, or level of support from their families/care partners). Further research is needed to define the targeted population which would most benefit and/or warrant the need for supports by a patient navigator, particularly to better understand the needs of women who perhaps live alone or are lonely, and may need additional help in accessing services and/or require counselling.

Since the PN Model of Care is novel, the most effective interventions and/or service remain unknown. In this retrospective observational cohort study, we described the PN program in a hospital setting in three areas (AC, ED, and RCC). PN models of care may be an ideal solution to minimize patients being readmitted to the hospital or designated as ALC by helping them navigate the system to find more appropriate supports in the community. In this model of care, the patient navigators work with clients using a person-centred approach to help them navigate the health and social care systems and overcome barriers to access services and resources.^26-28^ However, in this study, we did not include a comparative cohort which did not receive PN program. Future research should aim to capture the rates of hospital readmissions and ALC or delayed discharges, as well as to conduct a cost-effectiveness analysis to better determine the effectiveness of the PN program.

### PN Model of Care practice and implementation considerations

A contributing factor to the success of the PN program was the decision to embed the PN in the hospital, which facilitated communication^22^ between the patient navigators and the hospital staff (including in-hospital social workers) as they worked to help patients and family caregivers’ transition back home. The need for establishing clear and strong methods of communication among patient navigators, other healthcare providers, and patients has been identified as a critical factor for the successful implementation of PN programs.^29^ In our study, the embedded approach enabled patient navigators to effectively communicate more directly to hospital staff and patients about available community resources that were well-suited to patients’ needs, which potentially led to unnecessary hospitalization by enabling older adults to get access to community services.

One factor that facilitated this embedded approach was the formal relationship between the community agency and the hospital, established through a provincial healthcare initiative to strengthen working relationships among hospital and community agencies called Ontario Health Teams. Consequently, this new approach to healthcare delivery created the necessary conditions to have community-based patient navigators work in the hospital, which included the ability to participate in their care while patients were hospitalized. Although there are other examples of PN programs that use a similar embedded approach for other health conditions (e.g., cardiovascular disease^30^ and trauma patients^31^), to the best of our knowledge, none have done so for older adults with complex care needs.

Another critical factor related to the willingness of organizations to adopt PN programs is funding.^29^ In the present study, the PN program is being funded as a pilot project through a charitable donation from a foundation, and if not renewed, may hinder the sustainability of the program. This type of funding model for PN is common, whereby a scoping review on PN programs revealed that about a third of programs rely on grants, foundation support, or charitable donations.^29^ Hence, obtaining data to demonstrate the impact of the PN model of care on healthcare efficiency and patient outcomes is required to help advocate for long-term funding to sustain the program past the pilot phase.

## Conclusion

This research provided preliminary findings for a new PN Model of Care to support older adults in transitions in care who are being treated by a hospital's AC units, ED, and RCC. The patient navigators are social workers or “community transitional leads” who are involved with discharge planning, service and provider connections, housing alternatives, instrumental activities of daily living, and counselling. In this study, most patients did return to a home or supportive setting; however, further research is warranted with a comparative cohort. Since the patient navigator role is novel in a hospital setting, the study findings provide important insights and considerations for policy-makers and health leaders who are interested in implementing this PN Model of Care to improve transitions of care for older adults.

### Considerations for policy-makers and health leaders

1. This study provides preliminary evidence for implementing PN in a hospital.
2. Patient navigators are community social workers embedded in the hospital to facilitate transitions of care and to support older adults, their families, and/or care partners by providing follow-up care up to 90 days.
3. In addition to discharge planning, the patient navigators were also involved with service and provider connections, housing alternatives, instrumental activities of daily living, and counselling.
4. The patient navigators worked with the hospitals’ teams and supported a majority of the patients in returning home. Further research is warranted to further evaluate this care model.
